# Protein Corona Composition of Gold Nanocatalysts

**DOI:** 10.1021/acsptsci.4c00028

**Published:** 2024-03-14

**Authors:** Ali Akbar Ashkarran, Soheyl Tadjiki, Zijin Lin, Kylie Hilsen, Noor Ghazali, Sarah Krikor, Shahriar Sharifi, Meisam Asgari, Michael Hotchkin, Adam Dorfman, Karen S. Ho, Morteza Mahmoudi

**Affiliations:** † Department of Radiology and Precision Health Program, Michigan State University, East Lansing, Michigan 48824, United States; ‡ Postnova Analytics Inc., Salt Lake City, Utah 84102, United States; § Department of Medical Engineering, University of South Florida, Tampa, Florida 33620, United States; ∥ Clene Nanomedicine, Inc., Salt Lake City, Utah 84121, United States

**Keywords:** CNM-Au8, gold nanocatalysts, protein corona, blood–brain barrier, neurodegenerative diseases

## Abstract

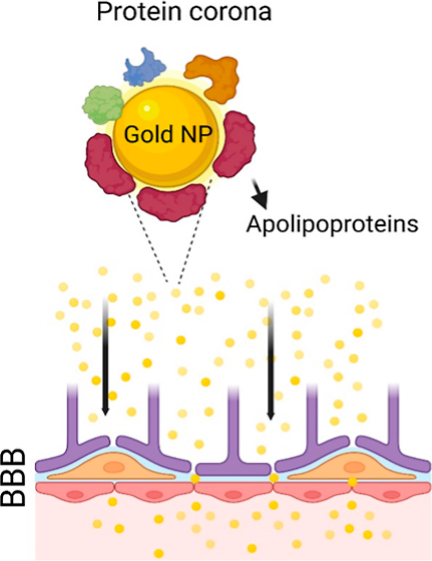

The interaction between nanoparticles (NPs) and biological environments is profoundly influenced by a stable, strongly adsorbed “hard” protein corona. This corona significantly determines the NPs’ pharmacokinetics and biological destiny. Our study delves into the mechanisms by which colloidal Au nanocrystals that are synthesized electrochemically without surface-capping organic ligands, known as CNM-Au8, traverse the blood–brain barrier (BBB) and target human brain tissue for treating neurodegenerative disorders. We discovered that upon interaction with human plasma, CNM-Au8 gold nanocrystals (AuNCs) effectively attract a variety of crucial apolipoproteins, notably apolipoproteins E, to their surfaces. This interaction likely facilitates their passage through the BBB. Furthermore, the coronas of these AuNCs exhibit a substantial presence of albumin and a notable absence of opsonin-based proteins, contributing to prolonged blood circulation. These characteristics align well with the clinical performance observed for the CNM-Au8 NCs. This study highlights that AuNCs with intentionally engineered structures and surfactant-free surfaces can create a distinct protein corona composition. This finding holds significant promise for the development of advanced therapeutic agents aimed at combating neurodegenerative diseases.

Gold nanoparticles (AuNPs) have earned acclaim for their adaptability and broad application in diagnostic and therapeutic nanomedicine.^1−3^ Their popularity stems from the simplicity of the synthesis process, the ability to control their shape, and the ease of surface functionalization.^4−7^ These characteristics, combined with their distinctive optical properties and inherent biocompatibility, make AuNPs a valuable tool in the field of nanomedicine.^4−11^

CNM-Au8, a registered trademark of Clene Nanomedicine, Inc., represents the first gold nanocrystal (AuNC) suspension being actively developed as a therapeutic drug for neurodegenerative disorders, including multiple sclerosis (MS), amyotrophic lateral sclerosis (ALS), and Parkinson’s disease (PD).^12−14^ This investigational nanomedicine has been evaluated in several completed Phase 2 clinical trials (REPAIR-MS: NCT03993171, VISIONARY-MS: NCT03536559, NCT04626921; RESCUE-ALS: NCT04098406; HEALEY-ALS Platform trial: NCT04414345; REPAIR-PD: NCT03815916).^15^ Preclinical studies have demonstrated the ability of orally administered CNM-Au8 to cross the blood–brain barrier (BBB), achieving therapeutic concentrations in the central nervous system,^13^ thus facilitating remyelination and improving functional/behavioral phenotypes.^13^ Correspondingly, initial^31^ phosphorus magnetic resonance brain imaging results from clinical trials involving participants with PD or relapsing MS have confirmed the ability of CNM-Au8 to penetrate the BBB, mirroring the results observed in preclinical models.^14^ Furthermore, CNM-Au8’s safety, tolerability, and biocompatibility have been established through numerous Phase 2 clinical trials, a first-in-human study, and compliance with ICH M3 (R2) standards for chronic toxicity in animals.

A key factor contributing to the therapeutic clinical success of CNM-Au8 lies in its novel synthesis approach. Unlike traditional methods of producing colloidal AuNPs, which rely on surface-capping organic ligands for crystal growth and stability, CNM-Au8 is synthesized without these ligands. This unique property not only ensures clinical safety and tolerability in human subjects but also enhances its biocompatibility profile, as evidenced by comprehensive safety evaluations in multiple Phase 2 clinical trials, first-in-human studies, and rigorous ICH M3 (R2) nonclinical toxicity testing.

It is widely recognized that the composition and arrangement of biomolecules, predominantly proteins, on the surface of NPs play a pivotal role in determining their pharmacokinetics and ultimate biological outcomes.^16−18^ To gain a deeper mechanistic insight into why CNM-Au8 AuNCs exhibit prolonged blood circulation time and the ability to cross the BBB, in this paper, we conducted a detailed investigation of their protein corona profiles.

## Results and Discussion

**Figure 1:**
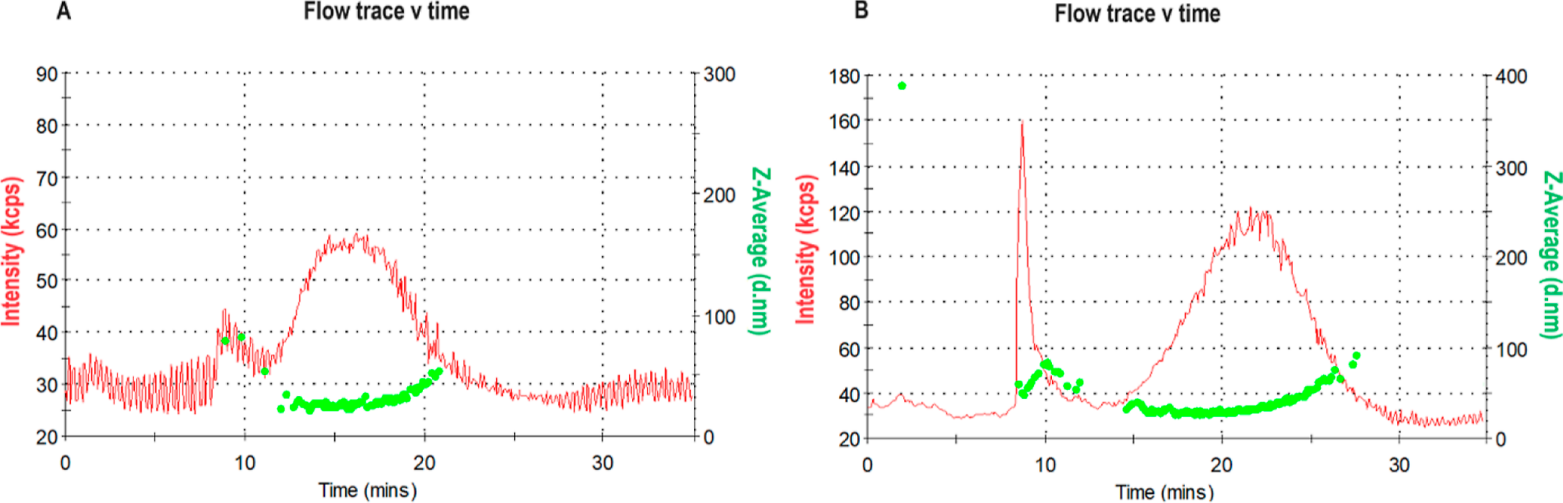
FFF results of CNM-Au8 AuNCs at a concentration of 10 μg/mL, suspended in (A) 6.5 mM NaHCO3 and (B) a buffered solution, which demonstrated an increase in the detected size of NPs indicative of aggregation.

We commenced our analysis of CNM-Au8 AuNCs by employing field-flow fractionation (FFF), dynamic light scattering (DLS), and zeta potential measurements. Table 1 consolidates the average measurements (based on five replicates) including the size of CNM-Au8 AuNCs in phosphate-buffered saline (PBS), their surface charge, polydispersity index (PDI), and the corresponding standard deviation (SD). We have also performed the FFF technique to evaluate size variations of the CNM-Au8 AuNCs in their original (6.5 mM NaHCO_3_) and buffered solutions (Figure 1). Our results demonstrated that the dispersion or dilution of CNM-Au8 AuNCs in PBS resulted in aggregation, a phenomenon that became more pronounced with escalating concentrations of CNM-Au8 AuNCs.

**Table 1:** Compilation of the Average Size, PDI, and Zeta Potential of CNM-Au8 AuNCs in PBS, Based on Five Replicates

CNM-Au8 concentration (μg/mL)	size (nm)	SD (nm)	PDI	SD	zeta potential (mV)	SD (mV)
3	162.2	51.7	0.245	0.024	–30.6	0.09
30	782.8	59.4	0.421	0.096	–41.9	0.1

**Figure 2:**
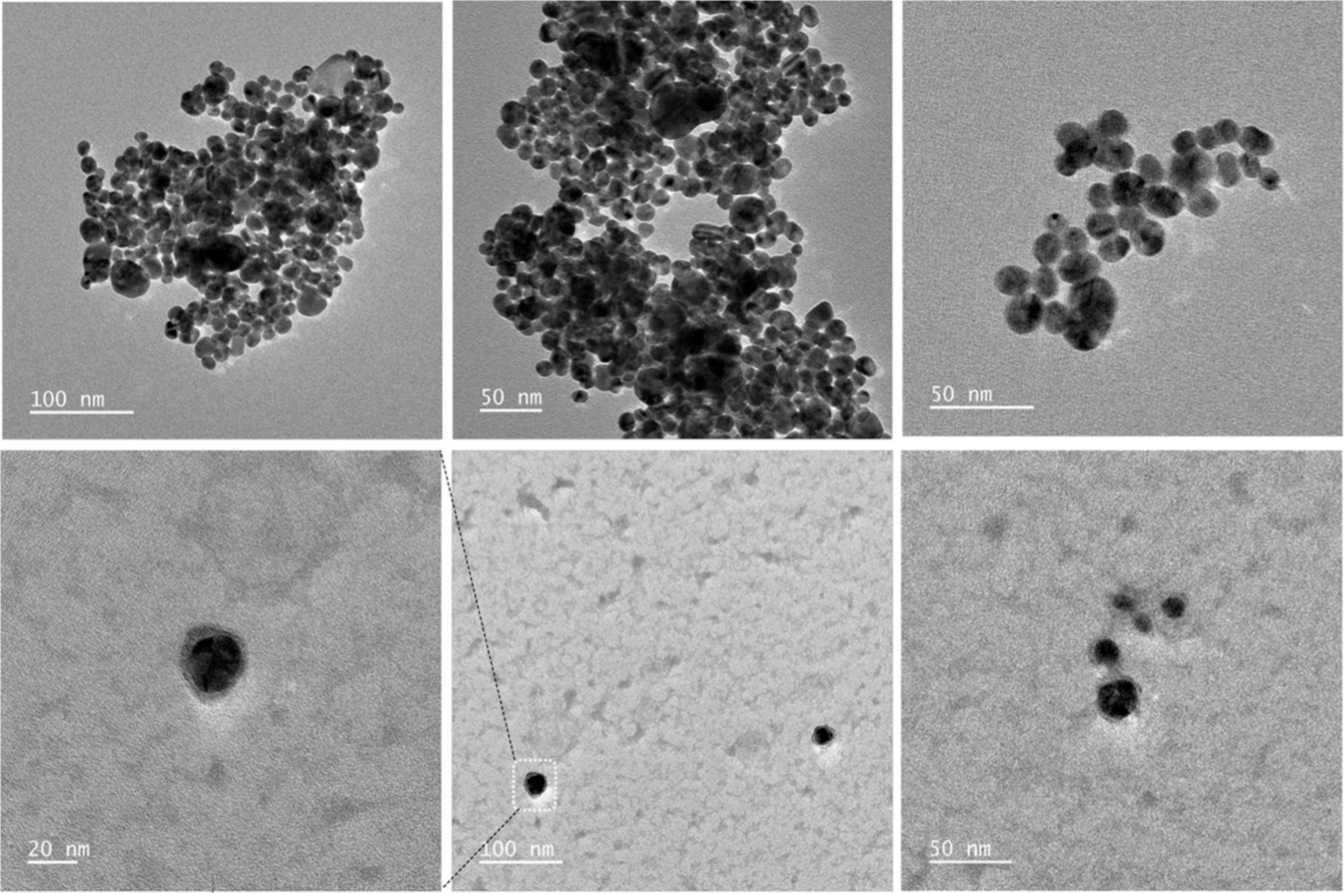
Representative TEM images of CNM-Au8 AuNCs prior to (top panels) and following (bottom panels) protein corona formation, highlighting the corona shell encasing the AuNCs’ surfaces.

**Figure 3:**
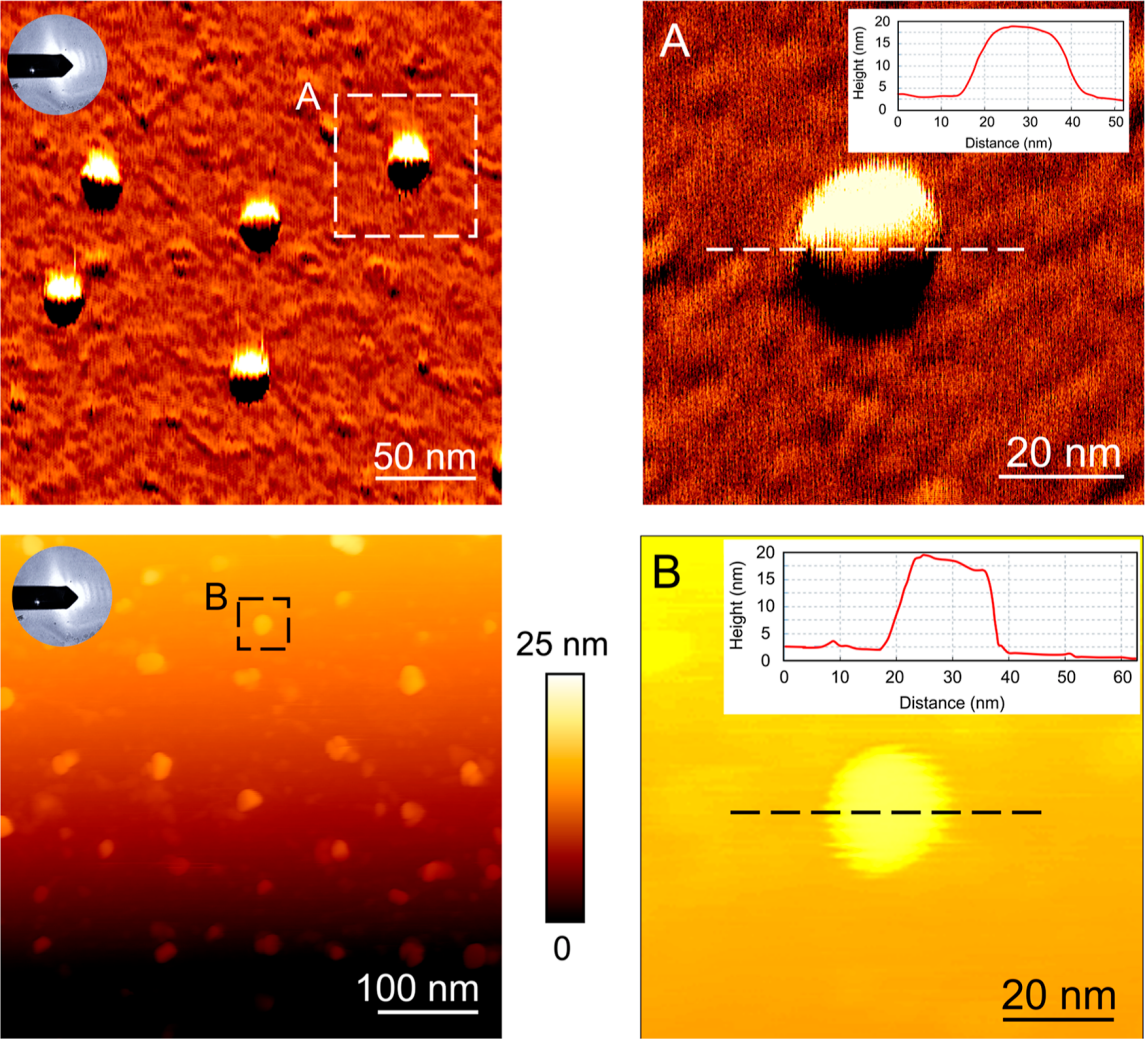
Representative AFM height and vertical deflection images showcase the uniform formation of corona-coated CNM-Au8 AuNCs. Insets A and B highlight the out-of-plane height of the selected particles, demonstrating the consistent coating achieved across the AuNCs.

The aggregation of the CNM-Au8 AuNCs that we observed is likely due to the absence of capping organic ligands on their surfaces. We theorized that creating a protein corona around these AuNCs could mitigate this issue as the resulting biomolecular layer would shield the CNM-Au8 AuNCs from aggregating. More specifically, as CNM-Au8 AuNCs can cross BBB, it is more likely that their surfaces are enriched with apolipoproteins, which in turn can improve the stability and solubility of AuNCs.^19^ For protein corona formation, three supraphysiologic concentrations of CNM-Au8 AuNCs (0.3, 1, and 3 μg/mL) were incubated with 55% human plasma [we used healthy human plasma from Innovative Research (www.innov-research.com) and diluted to a final concentration of 55% using PBS 1X] for 0.5, 1, 2, 12, and 24 h at 37 °C with constant agitation. CNM-Au8 concentrations were selected to provide a sufficient protein corona for analyses. To remove unbound and plasma proteins only loosely attached to the surface of CNM-Au8 AuNCs, protein-NP complexes were centrifuged in accordance with our previous optimization work using comparable NPs (see the Experimental Section for details).^20,21^ The fully washed protein corona-coated CNM-Au8 NCs were then used for robust protein characterization. The DLS analysis of these corona-coated AuNCs at selected incubation times (0.5 and 1 h) validated our hypothesis, demonstrating that the formation of a protein corona effectively reduces the aggregation of CNM-Au8 NCs regardless of their concentration and incubation time (Table 2). The lack of AuNCs’ aggregation is a crucial step for robust analysis of protein corona.^22^ We further conducted transmission electron microscopy (TEM) to visually show the formation of the protein corona at the surface of CNM-Au8 AuNCs (Figure 2). We conducted atomic force microscopy (AFM) analysis on corona-coated CNM-Au8 AuNCs, and the results (Figure 3) confirmed the formation of uniformly corona-coated NCs.

**Table 2:** Overview of Size Distribution, Zeta Potential, PDI, and Standard Deviations for Protein Corona-Coated CNM-Au8 AuNCs across Three Concentrations and Two Incubation Timepoints, Based on Five Replicates

CNM-Au8 concentration (μg/mL)	incubation time (h)	size (nm)	SD (nm)	PDI	SD	zeta potential (mV)	SD (mV)
0.3	0.5	39.1	10.5	0.457	0.035	–7.3	1.1
1		40.3	15.2	0.489	0.051	–10.7	1.8
3		37.4	13.9	0.508	0.055	–9.9	3.4
0.3	1	44.6	9.2	0.555	0.042	–9.7	1.9
1		42.4	2.4	0.498	0.024	–12.9	4.1
3		20.3	10.2	0.439	0.037	–9.9	1.9

**Figure 4:**
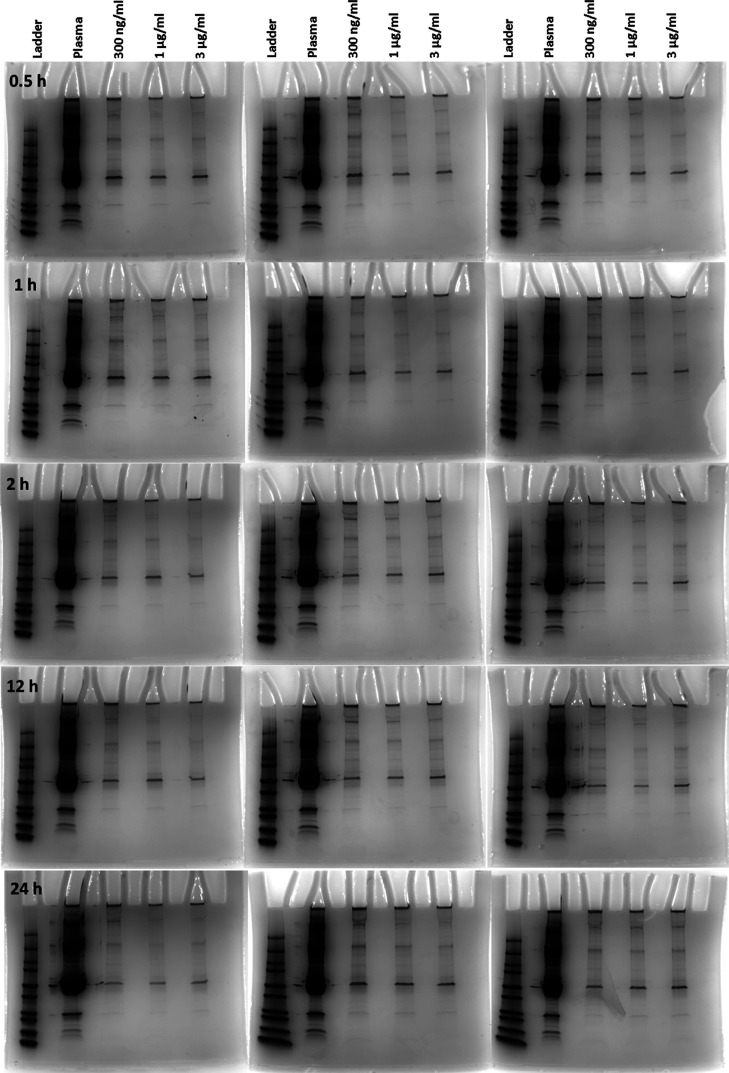
SDS-PAGE analysis of protein corona-coated CNM-Au8 NCs at different concentrations and incubation times. Each row represents images corresponding to replicates.

We conducted sodium dodecyl sulfate-polyacrylamide gel electrophoresis (SDS-PAGE) to examine the protein corona composition on CNM-Au8 AuNCs, with the findings illustrated in Figure 4. Our analysis revealed consistent protein corona patterns across different CNM-Au8 NC concentrations and incubation times, indicating high reproducibility in corona composition. To further identify and quantify the specific proteins in the CNM-Au8 NC protein corona, we employed liquid chromatography–mass spectrometry (LC–MS/MS). The comprehensive data from this analysis, including the CNM-Au8 NC protein corona profiles at varying concentrations and incubation temperatures, are detailed in Supplementary Excel Files S1–S16.

To calculate the percentage of specific proteins in the protein corona composition of CNM-Au8 AuNCs, we normalized protein abundances by molecular weight and expressed them as relative protein quantities. The calculation used the following formula
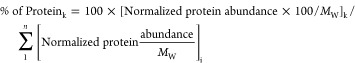
In this formula, *M*_w_ represents the molecular weight in kilodaltons (kDa) for protein k and n is the total number of proteins identified in the protein corona or plasma.

**Figure 5:**
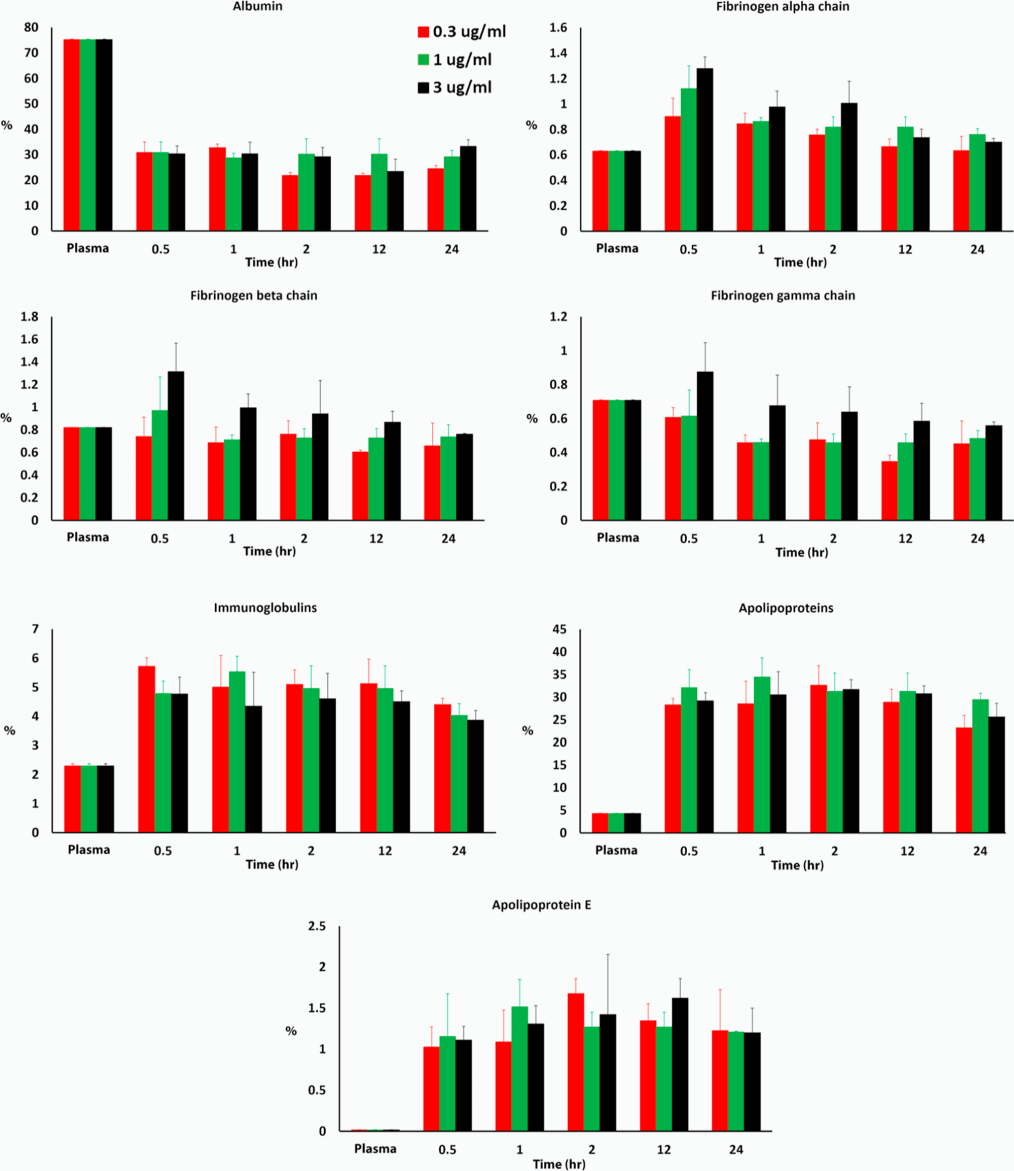
Percentage of albumin, different fibrinogen types, immunoglobulins, apolipoproteins, and apolipoprotein E in plasma versus protein corona profiles on CNM-Au8 NCs at various concentrations and incubation durations. Each bar represents the average of three separate experiments, each accompanied by its standard deviation. The graph demonstrates significant variations (*p* < 0.01) in albumin, immunoglobulins, and apolipoproteins when comparing their presence in plasma to their respective concentrations in the protein coronas of CNM-Au8 NCs at all concentrations and time points, underscoring the distinct interaction dynamics between these proteins and CNM-Au8 NCs.

The LC–MS/MS analysis of CNM-Au8 AuNCs unveiled three key insights into their biological identity, as depicted in Figure 5. First, the protein composition of the hard corona on CNM-Au8 AuNCs showed a selective binding pattern, deviating from plasma protein abundances. Notably, albumin’s percentage in plasma decreased from approximately 75% in the plasma control to about 30% in the CNM-Au8 AuNC protein corona.

Second, apolipoproteins were significantly enriched (by approximately 6-fold) in the corona of CNM-Au8 AuNCs, regardless of concentration and incubation time, compared to control plasma. This indicates a preferential binding of these proteins to the AuNCs’ surface. This is corroborated by the fact that other studies have also shown that the adsorption of apolipoproteins on AuNPs has been linked to the transport of AuNPs through BBB.^23,24^

In fact, apolipoproteins play a crucial role in facilitating the passage of AuNPs through the BBB through the following mechanisms: (i) certain apolipoproteins, such as ApoE, can bind to specific receptors on the BBB (e.g., low-density lipoprotein receptor-related protein) and therefore promote receptor-mediated transcytosis (i.e., a process where substances are transported across the endothelial cells of the BBB); (ii) apolipoproteins can mimic the low-density and/or high-density lipoproteins which are naturally transported across the BBB (e.g., reports have highlighted the significant role of apolipoprotein A-I in enhancing the uptake of protamine-oligonucleotide NPs^25^ and amyloid beta fibrils^26^ across the BBB) and, therefore, enhance the uptake and transport of CNM-Au8 AuNCs into the brain.^27−30^ Moreover, the substantial contribution of apolipoproteins to the coronas of CNM-Au8 AuNCs enhances the NCs’ colloidal stability and stealth effect, resulting in decreased nonspecific absorption by immune cells and consequently extending their circulation time in the blood.^31−33^ For example, analogous to the amphiphilic-like nature of apolipoproteins, which can encircle lipids resulting in the formation of a water-soluble lipoprotein particle, absorption of apolipoproteins on the surface of AuNPs can potentially provide a detergent-like surface coating as well as increase or enhance the stability of AuNPs, facilitating their transport through barriers such as BBB.^34^

Previous work by others has demonstrated that to attain a stealth effect, NPs can be precoated with apolipoproteins prior to in vivo administration^35^ or coated with specific compounds, such as poly(ethylene glycol) (PEG) or polyphosphoesters, to facilitate the adsorption of apolipoproteins during protein corona formation.^35,36^

Our results revealed (Figure 5 and Supplementary Excel files) that over 80% of the apolipoproteins participating in the corona composition of CNM-Au8 are comprised of five subtypes: apolipoproteins A-I, A-II, C-II, C-III, and E. These apolipoproteins, through direct or indirect mechanisms, enhance the NPs’ ability to cross the BBB. For instance, apolipoprotein A-I can traverse the BBB via clathrin-independent and cholesterol-mediated endocytosis.^37^ The interactions among these apolipoproteins can further enhance the penetration of NPs through the BBB. For instance, research has revealed that the presence of apolipoproteins A-I specifically bound to apolipoprotein C-III demonstrates a notable correlation with cerebrospinal fluid apolipoprotein C-III levels. In contrast, the correlation with total plasma apolipoprotein C-III is less pronounced.^38^ This observation suggests a preferential transport mechanism for apolipoprotein C-III across barrier interfaces, particularly when coexisting proteins like apolipoprotein A-I are present.^37,39^ This transport may occur through cellular-mediated mechanisms, clathrin-independent pathways, or cholesterol-mediated endocytosis.^37,39^

Third, the corona did not show a significant increase in complement proteins and fibrinogens compared to plasma. This suggests a reduced potential of NP eradication by the immune system, which in part, is due to the significant contribution of apolipoproteins to their corona profiles.^31−33^ More specifically, there were minor variations observed in the concentrations of different types of fibrinogens (such as an increase in the fibrinogen alpha chain) at the initial stages of incubation (Figure 5). However, these changes normalized to plasma levels over time, indicating a dynamic equilibrium of fibrinogen adsorption and desorption on the NPs’ surface. The lack of accumulated absorption of high amounts of immunoglobulin, and especially of fibrinogen chains, may be indicative of the absence of a fibrillation process that some proteins undergo after interaction with AuNPs.^40^

The CNM-Au8 NCs exhibited a distinct protein corona composition comprising a substantial presence of apolipoproteins (including apolipoprotein E) and a lower proportion of complement proteins alongside approximately 30% albumin. This unique composition potentially extends the bloodstream residency of CNM-Au8 NCs, thereby enhancing their chances of crossing the BBB, primarily aided by the high concentration of apolipoproteins.

## Conclusions

This study provides valuable insights into the protein corona composition of CNM-Au8 gold nanocatalysts and provides implications for CNM-Au8’s biomedical applications, particularly in the treatment of neurodegenerative disorders. The research revealed that CNM-Au8 NCs exhibit a unique protein corona, characterized by significant enrichment of apolipoproteins and a notable presence of albumin, while they largely lack opsonin-based proteins. This composition is crucial for the CNM-Au8 NCs’ enhanced stability, prolonged circulation in the bloodstream, and improved ability to cross the BBB. Our findings underscore the importance of protein corona composition in influencing the biological interactions and therapeutic efficacy of the NPs. The selective binding of apolipoproteins to CNM-Au8 AuNCs, particularly apolipoproteins A-I, A-II, C-II, C-III, and E, demonstrates their pivotal role in facilitating NPs’ transport through the BBB. These are critical factors for the therapeutic application of CNM-Au8 in targeting neurodegenerative diseases. Furthermore, the absence of significant levels of complement proteins and fibrinogens in the protein corona suggests a reduced immune response, contributing to the AuNCs’ extended residence time in the bloodstream. The innovative synthesis approach of CNM-Au8, free from surface-capping organic ligands, not only ensures safety and tolerability but also plays a key role in shaping its unique protein corona composition. This aspect is particularly relevant in the context of clinical applications, as evidenced by ongoing clinical trials. The distinctive protein corona of CNM-Au8 gold nanocatalysts opens new avenues for the development of NP-based therapies, particularly for neurodegenerative conditions, where BBB penetration is a significant challenge.

## Experimental Section

### Formation of Corona-Coated AuNCs and Their Physicochemical Characterization

To form protein corona-coated CNM-Au8 AuNCs, the AuNCs and human plasma (55% in PBS) were mixed and incubated at 37 °C for desired times and concentrations, followed by centrifugation at 17,000*g* for 30 min. The pellet was then resuspended in 0.5 mL of PBS and washed two more times to make sure all excess plasma was removed. The final pellets were redispersed in 50 μL of PBS and used for characterization. DLS and ζ-potential analyses were performed to measure the size distribution and ζ-potential of the AuNCs before and after protein corona formation using a Zetasizer nano series DLS instrument (Malvern Panalytical). A helium–neon laser with a wavelength of 632 nm was used for the size distribution measurement at room temperature (RT). TEM of NPs was carried out using a JEM-2200FS (JEOL Ltd.) operated at 200 kV. The instrument was equipped with an in-column energy filter and an Oxford X-ray energy-dispersive spectroscopy (EDS) system. 20 μL of the bare and protein corona-coated AuNCs was drop-cast onto a copper grid, followed by negative staining using 20 μL of 1% uranyl acetate, finally washed with DI water and used for imaging on the same day. Protein corona profiles of AuNCs were studied by SDS-PAGE analysis.

### FFF Methodology

In this study, we employed an asymmetrical flow FFF (AF4) system from Postnova Analytics GmbH, Landsberg, Germany. This system was connected to a comprehensive array of detectors, including (i) the PN3621 multiangle light scattering (MALS) detector; (ii) the PN3150 refractive index detector; (iii) the PN3211 UV–vis detector; and (iv) the Zetasizer Nano DLS detector from Malvern Instruments, Malvern, UK.

The AF4 apparatus was equipped with a standard separation channel, which had a thickness of 350 μm. In order to optimize the separation of CNM-Au8 AuNCs, we evaluated various membranes and ultimately chose a 10 kDa regenerated cellulose (RC) membrane for its efficacy. The carrier fluid consisted of a 0.05% (v/v) FL-70 solution provided by Thermo Fisher Scientific. A volume of 200 μL was introduced into the system at a flow rate of 0.2 mL/min by using an autosampler (PN5300, Postnova Analytics GmbH).

Prior to elution, the samples were subjected to a focusing phase at a flow rate of 1.8 mL/min for 7 min to ensure optimal separation. The separation of NC suspensions was achieved through a field-decayed method, which utilized a consistent channel flow rate and cross-flow rate of 1 mL/min each. Initially, the crossflow rate was kept constant for the first 2 min, and then it was gradually reduced to 0 mL/min over the course of 35 min, following a power equation with an exponent of 0.5, to efficiently separate the particles based on their size.

For the buffered solution, NPs were incubated with Dulbecco’s modified Eagle medium (DMEM) at 37 °C for a duration of 60 min.

### Atomic Force Microscopy

For imaging purposes, we also utilized a JPK NanoWizard 4 atomic force microscope from Bruker Nano, Berlin, Germany, which was integrated onto the stage of an inverted epifluorescence microscope, the Zeiss Axiovert 200M, provided by Carl Zeiss Microscopy, Göttingen, Germany. AFM was used to conduct lateral scans over areas varying from 50 μm × 50 μm to 500 × 500 nm at a scanning rate of 1 Hz, capturing images at a high resolution of 1024 × 1024 pixels. For these imaging tasks, we employed high-resolution Biotool qp-BioAC/Quartz cantilevers obtained from Nanotools USA LLC, Henderson, NV. These cantilevers feature a 2 nm defined conical tip, a length of 60 μm, a spring constant of 0.1 N/m, and a nominal resonance frequency of 50 kHz. All AFM imaging was performed under ambient RT conditions.

### LC–MS/MS Sample Preparation Methodology

#### Initial Processing

For protein corona-coated CNM-Au8 AuNCs, we initiated our protocol by washing them with PBS, followed by resuspension in 30 μL of PBS enhanced with 15 mM phosphate (pH 7.4). The total protein content bound to the particles was quantified as approximately 1 μg per sample, ascertained through a BCA assay.

Reagents and buffers employed:

- 250 mM ammonium bicarbonate (ABC)
- 100 mM ABC, serving as the digestion buffer (DB)
- 100 mM dithiothreitol (DTT) dissolved in DB
- Freshly prepared 100 mM iodoacetamide (IAA) in DB
- LysC/trypsin (Pierce-Thermo, cat# A41007) at a concentration of 1 μg/μL in storage buffer, subsequently diluted to 0.02 μg/μL in DB for use.

Sample processing protocol:

- Step 0: Initial sample in PBS (30 μL).
- Step 1: Introduction of DTT in DB (1 μL, 100 mM, achieving a final concentration of 2 mM), followed by incubation at 50 °C for 45 min with shaking at 700 rpm.
- Step 2: Addition of IAA in DB (4 μL, 100 mM, to reach a final concentration of 8 mM) and incubation at RT in darkness for 20 min.
- Step 3: Introduction of DTT in DB (4 μL, 100 mM, final concentration 8 mM) and incubation at RT for 15 min.
- Step 4: Addition of LysC/trypsin (5 μL, 0.02 μg/μL) and incubation at 37 °C overnight.

#### Post-digestion Processing

Upon completion of the digestion process, the samples were centrifuged at 16,000×*g* for 20 min at RT to segregate the NPs. The resulting supernatant, containing the peptide digest, was collected and subsequently desiccated using a SpeedVac. The samples then underwent desalting using Pierce C18 spin tips (Thermo, 84850), adhering to the manufacturer’s protocol, followed by another round of vacuum drying. The samples were stored in a refrigerator, pending LC–MS/MS analysis.

#### LC–MS/MS Analysis

For analysis, the dried samples were reconstituted with 1 μg of peptides in 25 μL of LC loading buffer (comprising 3% ACN and 0.1% TFA). We injected 5 μL aliquots of these samples in triplicate for LC/MS/MS analysis, employing a 60 min gradient in LFQ mode. Control samples, consisting of 55% human plasma, were prepared by mixing 8 μg of peptides in 200 μL of loading buffer and analyzed in triplicates. The analysis was conducted using an Ultimate 3000RSLCnano (Thermo Fisher) HPLC system with specific columns, solvents, and gradient settings. Data-dependent analysis (DDA) was performed with predefined MS and MS2 scan settings. Subsequent post-acquisition analysis was executed using Proteome Discoverer 2.4 (Thermo Fisher), following the specific search, identification, quantification, and validation parameters as documented in our previous publication (refer to center #9 for details).^27^

## Supporting Information Available

Information for the supplementary Excel file PDF
Comparative outcomes from three separate mass spectrometry analyses of the protein corona on CNM-Au8 nanocrystals at different concentrations and incubation times XLSX
