# Supplementary material for: Protein Corona Composition of Gold Nanocatalysts
Source: ACS Pharmacol Transl Sci. 2024 Mar 14;7(4):1169–77. doi: 10.1021/acsptsci.4c00028 (PMC11020068; doi:10.1021/acsptsci.4c00028)
Supplement: Supplementary file 1 — pt4c00028_si_001.pdf [file pt4c00028_si_001.pdf]

Supporting Information

Protein Corona Composition of Gold Nanocatalysts

Ali Akbar Ashkarran[1], Soheyl Tadjiki[2], Zijin Lin[1], Kylie Hilsen[1], Noor Ghazali[1], Sarah Krikor[1], Shahriar Sharifi[1], Meisam Asgari[3], Michael Hotchkin[4], Adam Dorfman[4], Karen S. Ho[4], and Morteza Mahmoudi[1*]

[1]Department of Radiology and Precision Health Program, Michigan State University, East Lansing, MI, 48824 USA

[2]Postnova analytics Inc., Salt Lake City, UT 84102, USA

[3]Department of Medical Engineering, University of South Florida, Tampa, FL, USA

[4]Clene Nanomedicine, Inc., Salt Lake City, Utah 84117, United States

***Corresponding author:** (MM) email: mahmou22@msu.edu

Information for the supplementary excel file:
S1: Comparative Outcomes from Three Separate Mass Spectrometry Analyses of the Protein Corona on CNM-Au8 Nanocrystals at a Concentration of 0.3 µg/ml Following a 0.5-Hour Incubation Period.

S2: Comparative Outcomes from Three Separate Mass Spectrometry Analyses of the Protein Corona on CNM-Au8 Nanocrystals at a Concentration of 1 µg/ml Following a 0.5-Hour Incubation Period.

S3: Comparative Outcomes from Three Separate Mass Spectrometry Analyses of the Protein Corona on CNM-Au8 Nanocrystals at a Concentration of 3 µg/ml Following a 0.5-Hour Incubation Period.

S4: Comparative Outcomes from Three Separate Mass Spectrometry Analyses of the Protein Corona on CNM-Au8 Nanocrystals at a Concentration of 0.3 µg/ml Following a 1-Hour Incubation Period.

S5: Comparative Outcomes from Three Separate Mass Spectrometry Analyses of the Protein Corona on CNM-Au8 Nanocrystals at a Concentration of 1 µg/ml Following a 1-Hour Incubation Period.

S6: Comparative Outcomes from Three Separate Mass Spectrometry Analyses of the Protein Corona on CNM-Au8 Nanocrystals at a Concentration of 3 µg/ml Following a 1-Hour Incubation Period.

S7: Comparative Outcomes from Three Separate Mass Spectrometry Analyses of the Protein Corona on CNM-Au8 Nanocrystals at a Concentration of 0.3 µg/ml Following a 2-Hours Incubation Period.

S8: Comparative Outcomes from Three Separate Mass Spectrometry Analyses of the Protein Corona on CNM-Au8 Nanocrystals at a Concentration of 1 µg/ml Following a 2-Hours Incubation Period.

S9: Comparative Outcomes from Three Separate Mass Spectrometry Analyses of the Protein Corona on CNM-Au8 Nanocrystals at a Concentration of 3 µg/ml Following a 2-Hours Incubation Period.

S10: Comparative Outcomes from Three Separate Mass Spectrometry Analyses of the Protein Corona on CNM-Au8 Nanocrystals at a Concentration of 0.3 µg/ml Following a 12-Hours Incubation Period.

S11: Comparative Outcomes from Three Separate Mass Spectrometry Analyses of the Protein Corona on CNM-Au8 Nanocrystals at a Concentration of 1 µg/ml Following a 12-Hours Incubation Period.

S12: Comparative Outcomes from Three Separate Mass Spectrometry Analyses of the Protein Corona on CNM-Au8 Nanocrystals at a Concentration of 3 µg/ml Following a 12-Hours Incubation Period.

S13: Comparative Outcomes from Three Separate Mass Spectrometry Analyses of the Protein Corona on CNM-Au8 Nanocrystals at a Concentration of 0.3 µg/ml Following a 24-Hours Incubation Period.

S14: Comparative Outcomes from Three Separate Mass Spectrometry Analyses of the Protein Corona on CNM-Au8 Nanocrystals at a Concentration of 1 µg/ml Following a 24-Hours Incubation Period.

S15: Comparative Outcomes from Three Separate Mass Spectrometry Analyses of the Protein Corona on CNM-Au8 Nanocrystals at a Concentration of 3 µg/ml Following a 24-Hours Incubation Period.
